# Brain Metastases from Ovarian Carcinoma

**DOI:** 10.5402/2011/527453

**Published:** 2011-12-01

**Authors:** Ettie Piura, Benjamin Piura

**Affiliations:** ^1^Department of Obstetrics and Gynecology, Sapir Medical Center, Sackler School of Medicine, University of Tel-Aviv, Kfar-Saba 44281, Israel; ^2^Unit of Gynecologic Oncology, Department of Obstetrics and Gynecology, Soroka Medical Center and Faculty of Health Sciences, Ben-Gurion University of the Negev, Beer-Sheva 84101, Israel

## Abstract

This paper will focus on knowledge related to brain metastases from ovarian carcinoma. So far, less than 600 cases were documented in the literature with an incidence among ovarian carcinoma patients ranging from 0.29% to 11.6%. The ovarian carcinoma was usually an advanced-stage epithelial serous carcinoma, and the median interval between diagnosis of ovarian carcinoma and brain metastases was 2 years. Most often, brain metastases, affected the cerebrum, were multiple and part of a disseminated disease. Treatment of brain metastasis has evolved over the years from whole brain radiotherapy (WBRT) only to multimodal therapy including surgical resection or stereotactic radiosurgery followed by WBRT and/or chemotherapy. The median survival after diagnosis of brain metastases was 6 months; nevertheless, a significantly better survival was achieved with multimodal therapy compared to WBRT only. It is suggested that brain imaging studies should be included in the followup of patients after treatment for ovarian carcinoma.

## 1. Introduction

The brain, along with the bone, liver, and lung, is one of the most common sites of metastasis with about 170,000 new cases of brain metastases diagnosed each year in the USA, a figure which is 10-fold higher than that of patients diagnosed with primary brain malignancies [1–3]. Common sources of brain metastases are lung, breast, renal and colorectal carcinoma, and malignant melanoma, and it has been estimated that up to 40% of patients with these cancers may develop brain metastasis in the course of their disease [1, 3–5]. Nevertheless, brain metastases from cancers of the female genital tract, apart from choriocarcinoma, are rare and usually found in association with widely disseminated systemic disease. The primary mechanism of spread from the genital tract to the brain is by hematogenous dissemination of tumor cells to the lungs and then to the brain via the pulmonary vasculature [6]. Ogawa et al. [6] reviewed 2,729 women with primary genital tract malignancies treated during 1985–2006 and identified 18 women (0.7%) with brain metastases. The incidence of brain metastases from ovarian carcinoma (7/335, 2.1%) was higher than those from uterine corpus carcinoma (4/556, 0.7%), uterine cervix carcinoma (7/1716, 0.4%), and other female genital tract malignancies combined (vagina, vulva, and fallopian tube carcinoma) (0/122, 0%).

In USA, epithelial ovarian carcinoma accounts for 3% of all cancers in women and is the ninth most common cancer (after breast, lung, colorectal, uterine corpus, thyroid, non-Hodgkin lymphoma, melanoma, and renal carcinoma) in women, with 21,990 estimated new cases in 2011 [7]. Ovarian carcinoma is responsible for 6% of all cancer deaths in women and is the fifth most common cause of death from cancer (after lung, breast, colorectal, and pancreas carcinoma) in women, with 15,460 estimated deaths from this disease in 2011 [7]. Ovarian carcinoma is the first most common cause of death from female genital tract malignancies in the western world and the second most common cause of death (after uterine cervix carcinoma) from female genital tract malignancies worldwide [8–10]. In 2007, the incidence (new cases/100,000 women/year) of ovarian carcinoma and the death rate (deaths/100,000 women/year) from ovarian carcinoma in USA were 12.5 and 8.2, respectively [11]. The National Cancer Institute (NCI) has estimated that 1.4% (1/70) of women born today in USA will develop epithelial ovarian carcinoma at some time in their lives [11]. In 2008, worldwide, 225,000 women were newly diagnosed with ovarian carcinoma (3.7% of all cancers in women), and 140,000 women died of this disease (4.2% of all deaths from cancer in women). Worldwide, ovarian carcinoma is the seventh most common cancer in women (after breast, colorectal, uterine cervix, lung, gastric, and uterine corpus carcinoma) and the seventh most common cause of death from cancer in women (after breast, lung, colorectal, uterine cervix, gastric, and liver carcinoma). The estimated incidence and death rate of ovarian carcinoma in 2008 worldwide have been 6.3 and 3.8, respectively [10].

Brain metastasis from ovarian carcinoma is uncommon, with less than 600 cases documented to date in the literature. Because of the rarity of brain metastasis from ovarian carcinoma, most reports include singular cases or series of patients in which patient accrual occurred over prolonged periods of time during which treatment approaches and modalities changed. This paper will focus on the following topics related to brain metastases from ovarian carcinoma: incidence of brain metastasis from ovarian carcinoma, characteristics and treatment of the primary ovarian carcinoma, interval between diagnosis of ovarian carcinoma and brain metastasis, type and site of brain metastasis, symptoms and signs of brain metastasis, CA-125 at diagnosis of brain metastases, prognostic indices for patients with brain metastases, treatment of brain metastases, and survival after diagnosis of brain metastases.

## 2. Incidence

Since the advent of chemotherapy some fifty years ago, an increase in the incidence of brain metastasis from various cancers has been noticed compared to the prechemotherapy era. This has been explained by the longer survival of cancer patients due to more aggressive treatments of the primary tumor, rising incidence of lung carcinoma and malignant melanoma, and ability to detect small tumors in the brain due to improvement in imaging techniques (e.g., computerized tomography [CT], magnetic resonance imaging [MRI], and positron emission tomography [PET]) [1, 3].

With the advent of platinum-based chemotherapy as standard treatment for epithelial ovarian carcinoma some three decades ago, a prolongation of survival of ovarian carcinoma patients compared to the preplatinum-based chemotherapy era has been achieved. Though more than 75% of patients with epithelial ovarian carcinoma are diagnosed with an advanced-stage disease (FIGO stage III/IV), a long-term survival is achieved in 15% to 30% of these patients by debulking (cytoreductive) surgery followed by platinum-based chemotherapy. Epithelial ovarian carcinoma is a disease that remains locoregionally confined until late in its natural history, and hematogenous metastases are rare at presentation (16%), with the most common sites of metastatic spread being the pleural cavity (33%), liver (26%), and lung (3%) [12]. Prolongation of survival of epithelial ovarian carcinoma patients achieved due to platinum-containing chemotherapy has been claimed to be the main reason for the rising incidence of brain metastasis from ovarian carcinoma. It has been claimed that while chemotherapy for ovarian carcinoma is effective in controlling tumor cell deposits in the abdominal cavity and elsewhere except the central nervous system (CNS), it is much less effective in controlling tumor cell deposits in the CNS since the chemotherapy molecules have difficulties in crossing the blood-brain barrier (BBB) [1, 3, 4]. The BBB is a continuous endothelium with tight junctions and little pinocytic vesicle activity that lines the microvasculature of the brain and, thus, limits the entry of circulating macromolecules into the brain parenchyma [4]. Nonetheless, the BBB does not prevent the invasion of the brain parenchyma by circulating metastatic cells [1, 4]. Fidler [4] and Langley and Fidler [1] showed that metastatic cells in the brain parenchyma exploit homeostatic mechanisms to their own advantage and, thus, have suggested that future therapy of brain metastases should be directed against both the tumor cells (“seed”) and the brain microenvironment (“soil”).

Since in the prechemotherapy era the survival of patients with advanced-stage epithelial ovarian carcinoma was short, and there was not enough time for brain metastasis to develop, data with respect to brain metastasis from ovarian carcinoma in the prechemotherapy era are quite scarce and mostly based on autopsy series. Mayer et al. [13] surveyed 576 autopsies of ovarian carcinoma patients collected from five large autopsy series published in the literature before 1978 [14–18] and found only five (0.86%) patients having brain metastases from ovarian carcinoma. The same authors [13] reviewed 1,441 patients with brain metastases collected from nine clinical and autopsy series published in the literature before 1978 and revealed that ovarian carcinoma was the source of brain metastases in 14 (0.97%) patients. These authors [13] also reviewed 97 patients with leptomeningeal metastasis collected from three series published in the literature before 1978 and observed that the ovary was not the source of leptomeningeal metastasis in any of the patients. LeChevalier et al. [19] reviewed 120 patients with brain metastases from various primary tumors and found that ovarian carcinoma was the source of brain metastases in 4 patients (6.5%) only. Larson et al. [20] surveyed 4,456 patients with epithelial ovarian carcinoma treated at The University of Texas M. D. Anderson Hospital and Tumor Institute during 1944–1984 and revealed 13 (0.29%) patients with brain metastases; remarkably, no patients were identified as having brain metastases before 1968. Kolomainen et al. [21] surveyed 3,690 patients with epithelial ovarian carcinoma treated at the Royal Marsden Hospital during 1980–2000 and found 18 (0.5%) patients with brain metastasis. An increase in the incidence of brain metastases from epithelial ovarian carcinoma over time was observed; the incidence during 1980–1984 was 0.2%, 1985–1989—0%, 1990–1994—0.3%, and 1995–1999—1.3% (*P* < 0.001) [21]. Chen et al. [22] checked their hospital's computer database of ovarian carcinoma patients and no patient with brain metastases was found before 2000, but 10 (1.9%) of 539 ovarian carcinoma patients treated during 2000–2007 had brain metastases. Dauplat et al. [23] reviewed the records of 255 patients with epithelial ovarian carcinoma treated at the University of California Los Angeles (UCLA) Medical Center during 1973–1983 and observed that brain metastases developed in five patients (1.96%) while pleural metastases developed in 63 patients (24.7%), liver metastases in 24 patients (9.4%), lung metastases in 18 patients (7.1%), distant lymph node (beyond pelvic and para-aortic chains) metastases in 18 patients (7.1%), skin metastases in 9 patients (3.5%), pericardial metastases in 6 patients (2.4%), and bone metastases in four patients (1.6%). Overall, 97 (38%) of the 255 patients had developed extraperitoneal or parenchymal liver metastases; the 5 patients with brain metastases accounted for 5.2% of these 97 patients [23]. 

Thirty-eight clinical series of patients with CNS metastasis from ovarian carcinoma, totaling 521 patients, have been published in the literature from 1978 until to date [6, 13, 20–22, 24–56] (Table 1). In 29 series in which the number of ovarian carcinoma patients is available, 34,728 ovarian carcinoma patients were surveyed, and 413 (1.19%) of them were found to have CNS metastases [6, 20–22, 24–27, 29–31, 33–35, 37, 39–43, 45–49, 52–55] (Table 1). The incidence of CNS metastasis among ovarian carcinoma patients was <1% in 5 series [20, 21, 24, 47, 48], 1–1.9% in 10 series [22, 27, 33, 39, 41–43, 49, 54, 55], 2–2.9% in 8 series [6, 29, 31, 34, 35, 45, 46, 53], 3–3.9% in 3 series [37, 40, 52], and ≥4% in 3 series [25, 26, 30] (Table 1). The claim made by some authors that the incidence of brain metastases among ovarian carcinoma patients is rising rapidly and approaching 12% [30] is not supported by most series published in the literature showing that the incidence of brain metastasis among ovarian carcinoma patients is within the range of 1–3% (Table 1).

## 3. Primary Ovarian Carcinoma

Some authors questioned whether patients with brain metastases from ovarian carcinoma tend to be younger at the time of diagnosis of ovarian carcinoma than the general ovarian carcinoma population [13, 27]. Nonetheless, it has been observed that the median age at the time of diagnosis of ovarian carcinoma of patients with brain metastasis is very close to the median age at the time of diagnosis of ovarian carcinoma in the general ovarian carcinoma population [37]. In the series of patients with brain metastases from ovarian carcinoma published in the literature, the median age of the patients at the time of diagnosis of ovarian carcinoma ranged from 44 to 60 years [6, 13, 20–22, 24–56].

The vast majority of the patients (>80%) with brain metastases from ovarian carcinoma had at the time of diagnosis of the primary disease an advanced-stage (stage III/IV) serous epithelial ovarian carcinoma [57]. The commonest histologic grade was 3 (poorly differentiated) [57]. Initial treatment of the ovarian carcinoma consisted generally of debulking (cytoreductive) surgery including total abdominal hysterectomy, bilateral salpingo-oophorectomy, and omentectomy followed by adjuvant chemotherapy. The accrual of patients in the series published in the literature occurred over prolonged periods of time during which chemotherapy regimens for ovarian carcinoma evolved considerably during the years from melphalan, through cyclophosphamide, through combination of doxorubicin and cyclophosphamide, through combination of cisplatin, doxorubicin and cyclophosphamide (PAC) to present day platinum- and taxane-based combination chemotherapy. Chemotherapy employed for the primary ovarian carcinoma in the series and singular cases reported in the literature included the following regimens: (1) oral chemotherapy: melphalan (Alkeran) [13, 27, 47], chlorambucil (Leukeran) [24], hexamethylmelamine (Altretamine) [13]; (2) intraperitoneal chemotherapy: thiotepa [24], cytosine arabinoside [35], carboplatin [58], paclitaxel [58]; (3) intravenous chemotherapy: cyclophosphamide (Cytoxan, Endoxan) [13, 47], doxorubicin (Adriamycin) [37], cisplatin [24, 45, 58], paclitaxel (Taxol) [59], doxorubicin and cyclophosphamide [37], cisplatin and oral chlorambucil [24], cisplatin and oral hexamethylmelamine [24, 37], cisplatin and cyclophosphamide [22, 35, 37, 45, 60, 61], carboplatin and cyclophosphamide [62, 63], carboplatin and oral chlorambucil [60], cisplatin and paclitaxel [43, 47], carboplatin and paclitaxel [22, 45, 48, 52, 64–67], carboplatin and docetaxel (Taxotere) [68], cisplatin, doxorubicin and cyclophosphamide [25, 27, 30, 31, 35–37, 39, 45, 47, 50, 58, 69], carboplatin, doxorubicin, and cyclophosphamide [35], cisplatin, paclitaxel and ifosfamide [52], cisplatin, paclitaxel and anthracycline [52], cisplatin, doxorubicin, cyclophosphamide and oral hexamethylmelamine [45, 70], “platinum-based chemotherapy” [38, 43, 44, 51, 55, 71].

A considerable number of patients treated for their primary ovarian carcinoma with platinum-based adjuvant chemotherapy were chemotherapy-sensitive and experienced a complete clinical response. Second-look procedure (laparoscopy/laparotomy) was performed in a portion of these patients and was reported to be negative in approximately half of the patients in whom it was performed [20, 25–27, 29, 31, 33, 38, 39, 43, 44, 47, 61–63, 65, 69, 72–75].

## 4. Brain Metastases

### 4.1. Interval between Diagnosis of Ovarian carcinoma and Brain Metastasis

The interval between diagnosis of ovarian carcinoma and brain metastases was available in 31 series totaling 460 patients [13, 21, 22, 24–28, 30–33, 35–38, 41–45, 47–56]. The medians of the interval between diagnosis of ovarian carcinoma and brain metastases ranged from 11 to 46 months with a median of the medians of 24.3 months (Table 1). Interestingly, brain metastasis was detected at the time of the diagnosis of ovarian carcinoma in four patients [30, 33, 43, 47], and brain metastasis was diagnosed two weeks prior to the diagnosis of ovarian carcinoma in one patient [51]. Brain metastasis diagnosed simultaneously with the ovarian carcinoma was documented by Bakar and Tekkök [76], and brain metastasis detected three months and two weeks before the diagnosis of ovarian carcinoma was reported by Izquierdo et al. [74] and Matsunami et al. [77], respectively.

LeRoux et al. [33] observed that the interval between diagnosis of ovarian carcinoma and brain metastasis was five times shorter in stage III/IV ovarian carcinoma patients than in stage I/II ovarian carcinoma patients. Cohen et al. [47] reported that patients with poorly differentiated (grade 3) ovarian carcinoma had a median interval of 1.5 years between diagnosis of ovarian carcinoma and brain metastases, as opposed to a median interval of 4.73 years in patients with well and moderately differentiated (grades 1 and 2) ovarian carcinomas (*P* = 0.03). It has, thus, been accepted that advanced-stage and poorly differentiated ovarian carcinoma place the patient at greater risk for brain metastasis. The vast majority of the patients in the reported series had advanced-stage (stage III/IV) ovarian carcinoma and was given intravenous platinum-based combination chemotherapy as postoperative first-line adjuvant chemotherapy. Although most patients initially responded to the chemotherapy, including complete responses, the tumor usually recurred within two years of cytoreductive surgery and chemotherapy. The relatively wide median interval (two years) between the diagnosis of ovarian carcinoma and diagnosis of brain metastasis strengthens the assumption that prolongation of life of ovarian carcinoma patients due to the control of intra-abdominal disease by platinum-based chemotherapy provides sufficient time for brain metastasis to develop and become apparent.

### 4.2. Type and Site of Brain Metastasis

Type of central nervous system (CNS) metastasis with respect to whether the metastasis is confined to the CNS only or is part of a disseminated disease was available in 35 series totaling 504 patients [13, 20–22, 24, 26–38, 40–56]; 236 (46.8%) patients had isolated CNS metastasis (metastasis confined to the CNS only), and 268 (53.2%) patients had CNS metastasis as part of a disseminated disease that affects also extracranial sites (Table 2). Among patients with concomitant extracranial metastatic disease at diagnosis of brain metastasis, the most common sites of extracranial recurrence were the peritoneum (pelvis and/or abdomen), liver, lungs, and lymph nodes [49]. Amount of CNS metastases with respect to whether the metastasis is a single (solitary) brain metastases or multiple brain metastases or leptomeningeal disease was available in 33 series totaling 489 patients [13, 20–22, 24, 26–29, 31–34, 36–38, 40–56], 205 (41.9%) patients had a single (solitary) brain metastases, 269 (55%) patients had multiple brain metastases, and 15 (3.1%) patients had leptomeningeal disease (Table 2). Site of metastasis in the CNS was available in 35 series totaling 504 patients [13, 20–22, 24, 26–38, 40–56]; the brain parenchyma was the site of metastasis in 489 (97%) patients and the leptomeninges were the site of metastasis in 15 (3%) patients. Of the 489 patients with brain parenchyma metastasis, the cerebrum was the site of brain metastasis in 210 (42.9%) patients, cerebellum in 44 (9%) patients, cerebrum and cerebellum in 11 (2.3%) patients, and site in the brain parenchyma not specified in 224 (45.8%) patients (Table 2). Thus, CNS metastasis is located in the vast majority of the patients (97%) in the brain parenchyma and is part of a disseminated metastatic disease that affects also extracranial sites in a slightly more than half of the patients. Parenchymal brain metastases are usually multiple and located most often in the cerebrum.

### 4.3. Symptoms and Signs of Brain Metastases

Common presenting symptoms and signs of brain metastases from ovarian carcinoma included headache (~50% of patients), confusion, dizziness, decreased mental status, consciousness disturbance, general weakness, extremity weakness, gait disturbance, neurological motor deficit, hemiparesis, ataxia, visual disturbance, papilledema, incontinence, nausea, vomiting, speech impairment (Aphasis), parasthesias, syncope, and seizure [13, 21, 22, 26, 27, 29, 31–33, 36–39, 43–45, 47, 48, 50–52, 54, 55, 57–61, 63, 65, 67–69, 74, 76, 77]. Increased intracranial pressure due to brain edema caused by the growth of metastases in the brain parenchyma is the main reason for the headache, nausea, vomiting, and papilledema [45].

### 4.4. CA-125 at Diagnosis of Brain Metastases

Serum CA-125 level was found to be increased (>35 U/mL) at the time of diagnosis of brain metastases from ovarian carcinoma in a subset of patients. Ten of 15 patients (66.6%) with brain metastases from ovarian carcinoma reported by Anupol et al. [43] had an elevated serum CA-125 level. The median serum CA-125 level for all patients was 51 (range, 4–1,756) U/mL. Serum CA-125 level did not predict the length of survival after brain metastases. Interestingly, 7 of 8 (87%) patients with isolated brain metastases had elevated serum CA-125 level compared with only 3 of 7 (42%) patients with additional extracranial disease [43]. Eight of 10 patients (80%) with brain metastases documented by Chen et al. [22] had elevated serum CA-125 level. The median of serum CA-125 level for all patients was 115 (range, 16–1,214) U/mL. All seven patients with additional extracranial disease had elevated serum CA-125 level but only one of three patients with isolated brain metastases had elevated serum CA-125 level (137 U/mL) [22]. Cormio et al. [71] found seven (50%) of 14 patients in whom serum CA-125 level was examined at the time of diagnosis of single (solitary) brain metastases to have elevated serum CA-125 level (median, 178 U/mL; range, 47–897 U/mL). Intraperitoneal disease was present in addition to brain metastases in five of the seven patients, whereas serum CA-125 elevation was attributed only to brain metastases in the remaining two patients and these values returned to normal after surgical resection of the brain metastases [71]. Seven of 18 patients with brain metastases documented by Kumar et al. [45] had an elevated serum CA-125 level (median, 133 U/mL; range, 116–800 U/mL). Of 15 patients with brain metastases from ovarian carcinoma treated by either gamma-knife radiosurgery (GKS) or whole-brain radiotherapy (WBRT) studied by Lee at al. [54], only 6 patients had increased CA-125 level (range, 42–3,700 U/mL) at diagnosis of brain metastases. Moreover, CA-125 level did not predict the survival rate after brain metastases (*P* = 0.52) [54]. In a series of 14 patients with brain metastases from ovarian carcinoma documented by Pothuri et al. [44], serum CA-125 levels prior to craniotomy were available in seven patients, and in five of these seven patients the levels were elevated (>35 U/mL). Of eight patients with brain metastases from ovarian carcinoma documented by Kastritis et al. [52], serum CA-125 level was elevated in two (25%) patients at the time of diagnosis of brain metastases. In four patients with brain metastases from ovarian carcinoma reviewed by Tay and Rajesh [48], CA-125 level at the time of diagnosis of brain metastases was 8.9, 44, 45.1, and 263 U/mL, respectively. Thus, it has been concluded that serum CA-125 is an unreliable marker for brain metastasis [48]. 

### 4.5. Prognostic Indices

Four prognostic indices have been designed for patients with brain metastases to guide treatment decisions [2]: (1) the *radiation therapy oncology group (RTOG) recursive partitioning analysis (RPA)* divides patients into three classes. Class I: Karnofsky performance status (KPS) ≥70, age <65 years, controlled primary tumor and no systemic disease; Class II: KPS ≥70 and at least one of these: age ≥65 years, uncontrolled primary tumor, presence of systemic disease; Class III: KPS <70 [78, 79]. (2) the *score index for radiosurgery (SIR)* is the sum of scores (0–2) for five prognostic factors: age (≥60, score 0; 51–59, score 1; ≤50, score 2), KPS (≤50, score 0; 60–70, score 1; 80–100, score 2), systemic disease (progressive, score 0; stable, score 1; complete response or no evidence of disease, score 2), number of brain metastases (≥3, score 0; 2, score 1; 1, score 2), and volume of largest brain metastases (>13 mL, score 0; 5–13 mL, score 1; <5 mL, score 2) [80]. (3) the *basic score for brain metastases (BSBM)* is the sum of scores (0-1) for three prognostic factors: KPS (50–70, score 0; 80–100, score 1), control of primary tumor (no, score 0; yes, score 1), and extracranial metastases (yes, score 0; no, score 1) [81]. (4). The *Graded Prognostic Assessment (GPA)* is the sum of scores (0, 0.5, and 1) for four prognostic factors: age (>60, score 0; 50–59, score 0.5; <50, score 1), KPS (<70, score 0; 70–80, score 0.5; 90–100, score 1), number of brain metastases (>3, score 0; 2-3, score 0.5; 1, score 1), and extracranial metastases (present, score 0; none, score 1) [2].

Of these prognostic indices, only the Radiation Therapy Oncology Group Recursive Partitioning Analysis (RTOG RPA) classification system has been applied by some authors to patients with brain metastases from ovarian carcinoma. Chen et al. [51] retrospectively reviewed the medical records of 19 patients with brain metastases from ovarian carcinoma treated at the Cleveland Clinic Foundation from 1985 to 2002. Overall, the median survival after diagnosis of brain metastases was 16.3 (range, 0.2–111.5) months. Retrospective application of the RTOG RPA classification system to these patients revealed a trend of survival advantage for patients allocated Class I (24.7 months) compared with patients allocated Class II (8.9 months) and Class III (2.6 months) (*P* = 0.31). Moreover, univariate (*P* = 0.003) and multivariate (*P* = 0.03) analyses showed that patients with a single (solitary) brain metastases had a longer survival from diagnosis of brain metastases (41.9 months) than those with multiple brain metastases (6.2 months). Furthermore, women with controlled primary ovarian carcinoma in the peritoneal cavity survived significantly longer (18.3 months) than those with an uncontrolled primary ovarian carcinoma (2.7 months) on univariate (*P* = 0.006) and multivariate (*P* = 0.01) analyses [51]. The authors [51] concluded that in addition to the RTOG RPA prognostic classification system, the number of metastatic lesions in the brain should also be taken into consideration in determining the prognosis for patients with brain metastases from ovarian carcinoma. Patients with good prognoses, as determined by the RTOG RPA prognostic classification system and number of brain metastases, will often benefit markedly from aggressive treatment, especially surgical resection of brain metastases when appropriate [51].

Kim et al. [53] evaluated prognostic factors in 13 patients with brain metastases from epithelial ovarian carcinoma. Overall, the median survival after detection of brain metastases was 7 months. Class of RTOG RPA was a significant predictor for survival by univariate analysis. The median survival after diagnosis of brain metastases of five patients allocated Class I was 26 months compared to 4 months of four patients allocated Class II and 4 months of four patients allocated Class III (univariate analysis, *P* = 0.007). In multivariate analysis, RTOG RPA class was of borderline significance (*P* = 0.09) [53]. In another study by Kim et al. [82] the charts of 25 patients with brain metastases who had died within 3 months of craniotomy were reviewed. Only one of these patients had brain metastases from ovarian carcinoma. In this patient, although the primary ovarian tumor in the peritoneum was under control at the time of diagnosis of brain metastases, there was lung metastasis in addition to brain metastases and the KPS was <70; thus, she was allocated RTOG RPA Class III. Based on the very short survival after craniotomy of patients allocated RTOG RPA Class III, the authors questioned the worth of craniotomy in the presence of RTOG RPA Class III [82].

## 5. Treatment of Brain Metastases

Data with respect to treatment modality of brain metastases was available in 34 series totaling 520 patients [13, 21, 22, 24, 26–39, 41–56]; 182 (35%) patients had WBRT only (median survival, 4.5 months), 79 (15.2%)—surgery and WBRT (median survival, 17 months), 70 (13.5%)—WBRT and chemotherapy (median survival, 9.1 months), 69 (13.3%)—surgery, WBRT, and chemotherapy (median survival, 20 months), 26 (5%)—surgery only (median survival, 6.7 months), 20 (3.8%)—stereotactic radiosurgery (SRS) or gamma-knife radiosurgery (GKRS) (median survival, 18 months), 10 (1.9%)—chemotherapy only (median survival, 7.5 months), 7 (1.3%)—surgery and chemotherapy (median survival not available), and 57 (11%)—no treatment (steroids only) (median survival, 1.4 months). Thus, therapy of brain metastases with combination of surgery, WBRT and chemotherapy, or combination of surgery and WBRT or SRS/GKRS yielded better survival results (median survival of 20, 17, and 18 months, resp.) than therapy of brain metastases with surgery alone, WBRT alone, chemotherapy alone, WBRT and chemotherapy, and no treatment (median survival of 6.7, 4.5, 7.5, 9.1, and 1.4 months, resp.). Thus, apparently, the best survival after diagnosis of brain metastases was achieved with multimodal therapy including surgical resection of the brain metastases followed by WBRT (±chemotherapy) or with SRS/GKRS.

In the above-mentioned series [13, 21, 22, 24, 26–39, 41–56], nevertheless, patient accrual occurred over prolonged periods of time during which treatment approaches and modalities for brain metastases changed. Traditionally, patients with isolated (limited to the brain only) and single (solitary) brain metastases generally would undergo resection of the brain lesion by craniotomy followed by WBRT. For patients with multiple brain metastases, with or without extracranial disease, WBRT with or without chemotherapy has usually been performed. In series and singular case reports published in literature before 1997, stereotactic radiosurgery (SRS) or gamma-knife radriosurgery (GKRS) was yet not included in the treatment of brain metastases from ovarian carcinoma. Kawana et al. [83] described in 1977 a patient with isolated multiple brain metastases from ovarian carcinoma which were successfully treated by a multimodality approach including GKRS. Initially, craniotomy was performed in this patient with resection of right occipital and cerebellar lesions, but a left temporal lesion was inaccessible. This was followed by WBRT and chemotherapy and then GKRS for resection of the remaining tumor in the temporal region. This multimodality approach which included GKRS has produced complete remission of the multiple brain metastases for 21 months with good quality of life [83]. Pothuri et al. [44] reported 14 patients having craniotomy for brain metastases from ovarian carcinoma during 1989–2001. In 11 of these 14 patients, craniotomy was followed by WBRT. In one of the 11 patients, WBRT was followed by stereotactic radiosurgery (SRS). The authors [44] observed a relatively longer survival achieved with craniotomy followed by WBRT and, thus, concluded that craniotomy followed by adjuvant WBRT can provide better control of brain metastases than WBRT alone. Cormio et al. [71] surveyed 22 patients who had surgical resection of solitary brain metastasis from ovarian carcinoma. Following surgery, 17 received WBRT and 5 received systemic chemotherapy. The median survival after diagnosis of brain metastasis was 16 (range, 4–41) months. In comparison, the median survival for another 34 patients who were not deemed eligible for surgical resection was only 4 (range, 1–24) months. The authors [71] concluded that neurosurgical resection of brain metastasis from ovarian carcinoma is indicated in solitary lesions in the absence of systemic disease.

Cohen et al. [47] reviewed 72 patients with brain metastases from ovarian carcinoma treated during 1975–2001 and found 69 patients in whom the mode of therapy was available; 8 patients were treated by craniotomy alone, 12—craniotomy and WBRT, 1—craniotomy and SRS, 35—WBRT alone, 1—WBRT and SRS, 1—WBRT and chemotherapy, 3—chemotherapy alone, and 8—no treatment. The authors [47] showed that the combination of surgical resection, and WBRT resulted in a longer survival (median, 23.07 months) than did WBRT alone (median, 5.33 months) or surgery alone (median, 6.9 months) (*P* < 0.01). Chen et al. [51] reviewed 19 patients with brain metastases from ovarian carcinoma treated during 1985–2002 and revealed that 9 patients were treated by WBRT alone, 6—WBRT and surgery, 1—surgical resection alone, 2—surgery and SRS, and 1—SRS and WBRT. Patients who underwent resection of brain metastases had a longer median survival (33.7 months) than those who did not undergo surgery (7.4 months) (*P* = 0.006) [51]. In a series of 13 patients with brain metastases from ovarian carcinoma reported by Kim et al. [53], 12 patients had treatment for their brain metastases. Of these 12 patients, 1 patient had GKRS only, 1—GKRS, resection of brain lesion and WBRT, 1—GKRS and WBRT, 1—WBRT, chemotherapy and GKRS, 3—WBRT and GKRS, 2—WBRT only, and 3—WBRT and chemotherapy. In 7 patients who received treatment including GKRS, the median survival after diagnosis of brain metastases was 23 months, while the median survival of the others was 4 months (*P* = 0.003). Moreover, multivariate analysis showed that treatment modality (treatment including GKSR versus treatment not including GKSR) was the only significant predictor of survival (*P* = 0.04) while RTOG RPA class was of borderline significance (*P* = 0.09) [53]. Lee at al. [54] reviewed 18 patients with brain metastases from ovarian carcinoma treated during 1983–2005. Five patients (33.3%) had extracranial metastases in addition to brain metastases, and 5 patients (33.3%) had single (solitary) brain metastases. In 15 patients, brain metastasis was treated with gamma-knife radiosurgery (GKRS) (7 patients) or WBRT (8 patients). Overall, median survival after diagnosis of brain metastasis was 14 (1–59) months. Nevertheless, patients treated with GKRS had a longer survival (median, 29 months) than those treated with WBRT (median, 6 months) (*P* = 0.0061). The authors [54] concluded that (1) GKRS seems to be an effective modality for the control of brain metastases and (2) GKRS improves the overall survival of patients with brain metastases from epithelial ovarian carcinoma. Sehouli et al. [55] reviewed 74 patients with brain metastases from ovarian carcinoma treated during 1981–2008. Eleven patients had surgery alone for their brain metastases, 20—WBRT alone, 14—surgery and WBRT, 2—surgery and chemotherapy, 6—WBRTand chemotherapy, and 21—surgery, WBRT, and chemotherapy. Thus, multimodal therapy for brain metastases was applied in 43 (58.1%) of the patients, the most common modality being a combination of surgical resection, WBRT, and chemotherapy (21 patients, 28.4%). The most frequently applied monotherapy was WBRT (20 patients, 27%). The authors [55] could not show that the use of multimodal strategies for brain metastases translate into a significant prolongation of survival (Hazard Ratio, 0.57; 95% CI, 0.31–1.05) compared with monotherapy. Chen et al. [22] reviewed 10 patients with brain metastases from ovarian carcinoma treated during 2000–2007. Nine of the 10 patients received treatment for their brain metastases; 3 patients had WBRT alone, 4—WBRT followed by chemotherapy, 1—surgery followed by WBRT and chemotherapy, and 1—GKRS followed by chemotherapy. The authors [22] concluded that the goal of treatment of brain metastases is the alleviation of the neurological symptoms and improvement of the quality of life; nevertheless, they could not make any conclusion from their data regarding the effect of mode of therapy on survival.

Cormio et al. [56] investigated in 2011 the changes in the management and outcome of CNS involvement from ovarian carcinoma since 1994. They compared the clinical and pathologic characteristics, treatment, and outcome of 23 patients with brain metastases from epithelial ovarian carcinoma who were treated during 1982–1994 with those of 20 patients treated during 1995–2010. The main difference between the two groups was the therapeutic approach to brain metastases. During 1982–1994, most patients received WBRT only as follows: WBRT only—14 (60.9%), surgical resection, and WBRT—5 (21.7%), steroids only—4 (17.4%). During 1995–2010, the therapeutic approach was more aggressive; 50% of patients underwent surgical resection and most patients received multimodal treatment with adjuvant radiotherapy or chemotherapy as follows: surgery and WBRT—3 (15%), surgery, WBRT and chemotherapy—3 (15%), chemotherapy, and WBRT—3 (15%), surgery and chemotherapy—3 (15%), WBRT alone—3 (15%), surgery alone—1 (5%), chemotherapy alone—1 (5%), and steroids only—3 (15%). During 1982–1994, the median survival after diagnosis of brain metastases was 5.0 months only, whereas it was 17.6 months during 1995–2010 (*P* = 0.03). The authors [56] concluded that an aggressive multimodal treatment approach including surgical resection followed by radiotherapy and/or chemotherapy might prolong the survival of patients with brain metastases from ovarian carcinoma.

Although the experience of using SRS/GKRS in the treatment of brain metastases from ovarian carcinoma is still limited, it seems that SRS/GKRS will gain more popularity in the future since patients with a limited number of brain metastases may be treated with SRS/GKRS without WBRT. Repeat SRS/GKRS may be performed for new lesions in the brain to avoid or delay use of WBRT for as long as possible. The rationale for this approach is the avoidance of significant neurotoxicity from WBRT. Thus, patients treated with SRS alone may experience fewer cognitive and constitutional side effects. In addition, there is an advantage for use of SRS in treating patients with single brain metastases who are unable to tolerate surgery and for those with surgically inaccessible lesions. Kim et al. [53] have asserted that brain metastases from ovarian carcinoma are good candidates for SRS because of their spherical shape and, therefore, the role of SRS for the treatment of brain metastases from ovarian carcinoma is expected to increase.

## 6. Survival

The survival time after diagnosis of ovarian carcinoma was available in 16 series totaling 236 patients [13, 20, 24, 26, 27, 29–31, 35, 38, 43, 45, 47, 49, 52, 55] (Table 1). The medians of the survival times after diagnosis of ovarian carcinoma ranged from 24 to 67 months with a median of the medians of 33.3 months (Table 1). The survival time after diagnosis of CNS metastases was available in 36 series totaling 513 patients [6, 13, 20–22, 24, 26–38, 40–56] (Table 1). The medians of the survival times after diagnosis of brain metastases ranged from 1 to 28 months with a median of the medians of 6.4 months. Thus, overall, the survival of patients after diagnosis of brain metastases from ovarian carcinoma is poor. Nevertheless, the survival after diagnosis of brain metastases is affected by the status (controlled versus uncontrolled) and extent (cranial metastases only versus cranial and extracranial metastases) of the primary disease and by the number, volume, and site of metastases in the brain parenchyma. In patients in whom multimodal therapy containing craniotomy or SRS/GKRS is feasible, the use of multimodal therapy containing craniotomy or SRS/GKRS may increase considerably the survival after diagnosis of brain metastases from ovarian carcinoma.

## 7. Conclusion

Brain metastasis from ovarian carcinoma is uncommon with less than 600 cases documented in the literature. In the prechemotherapy era, the incidence of brain metastasis among living ovarian carcinoma patients was almost nil because of the short survival of advanced-stage ovarian carcinoma patients that did not allow enough time for brain metastasis to develop. With the advent of chemotherapy for ovarian carcinoma, especially platinum-based chemotherapy, a considerable lengthening in the survival of advanced-stage ovarian carcinoma patients with a median of about two years has been achieved. This prolonged survival has allowed sufficient time for brain metastases to develop and become apparent. Nevertheless, the notion expressed by some authors that the incidence of brain metastasis among ovarian carcinoma patients is continuing to rise in the postchemotherapy era and even approaching >10% is not supported by most series published in the literature that show that the incidence of brain metastases among ovarian carcinoma patients is usually within the range of 1–3%. The primary ovarian carcinoma metastasizing to the brain is usually an advanced-stage (III/IV) and high-grade (G3) epithelial serous carcinoma that was treated primarily by cytoreductive (debulking) surgery followed by adjuvant chemotherapy. Some of these patients had after completion of first-line chemotherapy a second-look procedure which was often negative. The median interval between diagnosis of ovarian carcinoma and detection of brain metastasis is about two years. Most often, brain metastase affecting the cerebrum, are multiple and part of a disseminated disease. The following variables affect the prognosis and guide treatment decisions in patients with brain metastases from ovarian carcinoma: age, Karnofsky performance status (KPS), state of control of the primary disease, absence or presence of extracranial metastases, the number, location, and volume of brain metastases, and mode of therapy of brain metastases. Treatment of brain metastasis has evolved over the years from WBRT only for most patients to multimodal therapy including surgical resection, if feasible, followed by WBRT and/or chemotherapy. The median survival after diagnosis of brain metastases is about 6 months; nevertheless, a better survival is achieved with multimodal therapy, if feasible, including surgical resection than with WBRT alone. The experience of using SRS or GKRS in the treatment of brain metastases from ovarian carcinoma is still limited. Early detection of brain metastases in ovarian carcinoma patients is of utmost importance since brain metastases at their early stage of development in the brain parenchyma are still of small volume and, thus, much more feasible for surgical resection or SRS/GKRS with less complications and better survival than metastases at an advanced stage of their development in the brain. Thus, it is suggested that brain imaging studies should be included in the routine followup of patients after primary treatment of ovarian carcinoma. Besides, the emergence of one of more neurological symptoms and signs in an ovarian carcinoma patient should prompt an immediate search for brain metastases with use of brain imaging studies.

## Figures and Tables

**Table 1:** Incidence of CNS metastases among ovarian carcinoma patients, interval between diagnosis of ovarian carcinoma and CNS metastases, survival after diagnosis of ovarian carcinoma, and survival after diagnosis of CNS metastases.

Author (year) [ref.]	Study period	Number of patients with OC	Number of patients with CNS metastases (%)	Median Interval between diagnosis of OC and CNS metastases in months (range)	Median survival after diagnosis of OC in months (range)	Median survival after diagnosis of CNS metastases in months (range)
Mayer et al. (1978) [13]	1973–1977	NA	6	26 (2–82)	38 (8–85+)	4.5 (2–33+)
Barker et al. (1981) [24]	1969–1979	430	4 (0.9)	34 (11–72)	37 (13–78)	4 (0.2–8)
Budd et al. (1983) [25]	1978–1980	42	3 (7.1)	19 (19–20)	NA	NA
Larson et al. (1986) [20]	1944–1984	4,456	13 (0.29)	NA	26	5
Stein et al. (1986) [26]	1979–1985	110	5 (4.5)	17 (16–43)	28 (18–43)	2 (1–11)
Dauplat et al. (1987) [27]	1973–1983	255	5 (1.96)	25 (10–126)	24 (11–135)	1 (1–10)
Ziegler et al. (1987) [28]	1983–1985	NA	5	11 (11–31)	NA	8 (1–9+)
Ross et al. (1988) [29]	1980–1984	342	7 (2)	NA	27 (12–52)	6.5 (2–26)
Hardy and Harvey (1989) [30]	1981–1984	52	6 (11.6)	28.5 (0–36)	30 (3–126)	4.5 (2–41+)
Piura et al. (1990) [31]	1961–1988	200	2 (1)	22 (21–23)	41.5 (40–43)	19.5 (19–20)
Plaxe et al. (1990) [32]	NA	NA	6	28.5 (2–61)	NA	10 (2–24)
LeRoux et al. (1991) [33]	1980–1989	1,316	14 (1.1)	14.5 (0–72)	NA	3 (0.1–36)
Rodriguez et al. (1992) [34]	1977–1990	795	16 (2)	NA	NA	9
Bruzzone et al. (1993) [35]	1981–1989	413	9 (2.2)	19 (3–36)	26 (10–81)	8 (1–45)
Salvati and Cervani (1994) [36]	1980–1988	NA	4	21 (10–26)	NA	28 (17–48)
Geisler and Geisler (1995) [37]	1979–1992	479	16 (3.3)	19 (2–41)	NA	3 (1–24)
Cormio et al. (1995) [38]	1982–1994	NA	23	35 (5–114)	42 (8–133)	5 (1–24)
Suzuki et al. (1999) [39]	1982–1998	311	4 (1.3)	NA	NA	NA
Zhao et al. (1999) [40]	1989–1997	132	4 (3%)	NA	NA	10 (2–19+)
Kaminsky-Forrett et al. (2000) [41]	1974–1998	704	8 (1.1)	15 (2–80)	NA	3 (1–12)
Sanderson et al. (2002) [42]	1995–2000	1,222	13 (1.1)	36 (6–60)	NA	6 (2–42+)
Anupol et al. (2002) [43]	1986–2000	1,042	15 (1.4)	22 (0–53)	38 (9–82)	6 (0–49)
Kolomainen et al. (2002) [21]	1980–2000	3,690	18 (0.5)	46 (12–113)	NA	7 (1–41)
Pothuri et al. (2002) [44]	1989–2001	NA	14	42 (15.6–98.4)	NA	18 (0.5–32.8)
Kumar et al. (2003) [45]	1991–2001	658	18 (2.7)	29 (0–101)	30.5 (5–110)	4 (1–74)
Li and Fu (2003) [46]	1996–2000	478	10 (2.1)	NA	NA	6.3 (<1–33)
Cohen et al. (2004) [47]	1975–2001	8,225	72 (0.9)	22 (0–219)	40.5 (95% CI, 21.5–60)	6.3 (95% CI, 5–8)
Tay and Rajesh (2005) [48]	1993–2003	605	4 (0.66)	16.5 (8–65)	NA	19.5
Pectasides et al. (2005) [49]	1983–2004	1,450	17 (1.17)	15.9 (1.4–70.8)	27.4 (3–71.4)	5.7 (0.2–22.6)
D'Andrea et al. (2005) [50]	1980–2000	NA	11	21	NA	28
Chen et al. (2005) [51]	1985–2002	NA	19	25.2 (−0.6–112)	NA	16.3 (0.2–111.5)
Kastritis et al. (2006) [52]	1995–2004	267	8 (3)	17.2	67 (95% CI, 48–85)	22 (95% CI, 12–34)
Kim et al. (2007) [53]	1996–2005	490	13 (2.7)	28 (13–99)	NA	7 (0–30)
Ogawa et al. (2008) [6]	1985–2006	335	7 (2.1)	NA	NA	7.3 (0.9–48.2)
Lee et al. (2008) [54]	1983–2005	1,413	18 (1.3)	28 (8–71)	NA	14 (1–63)
Sehouli et al. (2010) [55]	1981–2008	4,277	74 (1.7)	28.8 (2.6–133.1)	36.2 (95% CI, 33.3–39.1)	6.2 (95% CI, 4.9–7.5)
Chen et al. (2011) [22]	2000–2007	539	10 (1.9)	24.3 (7–55)	NA	3 (0–16)
Cormio et al. (2011) [56]	1995–2010	NA	20	33 (3–70)	NA	17.6 (0–59)

Total		34,728**^a^**	521**^b^** 413**^c^** (1.19)	24.3 (11–46)**^d^**	33.3 (24–67)**^d^**	6.4 (1–28)**^d^**

CNS: central nervous system; OC: ovarian carcinoma; NA: not available.

**^a^**This number represents the total number of patients with ovarian carcinoma in 29 series in which the number of ovarian carcinoma patients is available.

**^b^**This number represents the total number of patients with brain metastases from ovarian carcinoma in all 38 series reviewed.

**^c^**This number represents the total number (percentage) of patients with brain metastases from ovarian carcinoma in 29 series in which the number of ovarian carcinoma patients is available.

**^d^**Median (range) of the medians. Interval between diagnosis of ovarian carcinoma and brain metastases was available in 31 series totaling 460 patients. Survival after diagnosis of ovarian carcinoma was available in 16 series totaling 236 patients. Survival after diagnosis of brain metastases was available in 36 series totaling 513 patients.

**Table 2:** Type, amount and site of CNS metastases from ovarian carcinoma.

		Type and amount of CNS metastases				Site of metastases in the brain parenchyma			
		Number of patients (%)				Number of patients			
Author (year) [ref.]	Number of patients with CNS metastases	Isolated	Brain parenchyma single (solitary)	Brain parenchyma Multiple	LM disease	NS	Cerebrum	Cerebellum	Cerebrum and cerebellum
Mayer et al. (1978) [13]	6	1 (17)	3 (50)	1 (17)	2 (33)	0	3	1	0
Barker et al. (1981) [24]	4	1 (25)	3 (75)	1 (25)	0 (0)	0	2	2	0
Larson et al. (1986) [20]	13	5 (38)	5 (38)	7 (54)	1 (8)	12	0	0	0
Stein et al. (1986) [26]	5	4 (80)	2 (40)	2 (40)	1 (20)	4	0	0	0
Dauplat et al. (1987) [27]	5	1 (20)	3 (60)	2 (40)	0 (0)	0	4	1	0
Ziegler et al. (1987) [28]	5	2 (40)	3 (60)	2 (40)	0 (0)	1	1	3	0
Ross et al. (1988) [29]	7	3 (43)	4 (57)	2 (29)	1 (14)	0	5	1	0
Hardy and Harvey (1989) [30]	6	4 (67)	NA	NA	NA	6	0	0	0
Piura et al. (1990) [31]	2	1 (50)	2 (100)	0 (0)	0 (0)	0	2	0	0
Plaxe et al. (1990) [32]	6	5 (83)	0 (0)	5 (83)	1 (17)	0	0	4	1
LeRoux et al. (1991) [33]	14	6 (43)	9 (64)	5 (36)	0 (0)	0	14	0	0
Rodriguez et al. (1992) [34]	16	11 (69)	5 (31)	10 (63)	1 (6)	15	0	0	0
Bruzzone et al. (1993) [35]	9	2 (22)	NA	NA	NA	9	0	0	0
Salvati and Cervoni (1994) [36]	4	4 (100)	4 (100)	0 (0)	(0)	0	4	0	0
Geisler and Geisler (1995) [37]	16	8 (50)	8 (50)	8 (50)	0 (0)	0	14	2	0
Cormio et al. (1995) [38]	23	9 (39)	9 (39)	13 (56)	1 (4)	0	18	4	0
Zhao et al. (1999) [40]	4	2 (50)	3 (75)	1 (25)	0 (0)	4	0	0	0
Kaminsky-Forrett et al. (2000) [41]	8	1 (13)	6 (75)	2 (25)	0 (0)	0	3	3	2
Sanderson et al. (2002) [42]	13	5 (38)	4 (31)	8 (62)	1 (8)	0	10	2	0
Anupol et al. (2002) [43]	15	8 (57)	7 (47)	7 (47)	1 (7)	14	0	0	0
Kolomainen et al. (2002) [21]	18	8 (44)	9 (50)	9 (50)	0 (0)	18	0	0	0
Pothuri et al. (2002) [44]	14	7 (50)	12 (86)	2 (14)	0 (0)	0	11	3	0
Kumar et al. (2003) [45]	18	5 (28)	5 (28)	12 (67)	1 (5)	0	13	2	2
Li and Fu (2003) [46]	10	0 (0)	2 (20)	8 (80)	0 (0)	0	10	0	0
Cohen et al. (2004) [47]	72	31 (43)	25 (35)	44 (61)	3 (4)	0	63	6	0
Tay and Rajesh (2005) [48]	4	0 (0)	0 (0)	3 (75)	1 (25)	0	3	0	0
Pectasides et al. (2005) [49]	17	13 (76)	5 (29)	12 (71)	0 (0)	17	0	0	0
D'Andrea et al. (2005) [50]	11	11 (100)	11 (100)	0 (0)	0 (0)	0	9	2	0
Chen et al. (2005) [51]	19	9 (47)	7 (37)	12 (63)	0 (0)	19	0	0	0
Kastritis et al. (2006) [52]	8	2 (25)	4 (50)	4 (50)	0 (0)	0	6	2	0
Kim et al. (2007) [53]	13	6 (46)	2 (15)	11 (85)	0 (0)	0	6	1	6
Lee et al. (2008) [54]	15**^a^**	10 (67)	5 (33)	10 (67)	0 (0)	15	0	0	0
Sehouli et al. (2010) [55]	74	39 (52.7)	26 (35)	48 (65)	0 (0)	74	0	0	0
Chen et al. (2011) [22]	10	3 (30)	1 (10)	9 (90)	0 (0)	10	0	0	0
Cormio et al. (2011) [56]	20	9 (45)	11 (55)	9 (45)	0 (0)	6	9	5	0

Total	504	236 (46.8)	205 (41.9)**^b^**	269 (55)**^b^**	15 (3.1)**^b^**	224 (45.8)**^c^**	210 (42.9)**^c^**	44 (9)**^c^**	11 (2.3)**^c^**

CNS: central nervous system; LM: leptomeningeal; NS: not specified; NA: not available.

**^a^**There were 18 patients with BM; however, data were analyzed for 15 patients treated with gamma-knife surgery (GKS) or whole brain radiotherapy (WBRT).

**^b^**Percentage is calculated for 489 patients in whom amount and distribution of CNS metastases is available.

**^c^**Percentage is calculated for 489 patients with parenchymal brain metastases.
